# Reshaping Resistance: How Autovaccine Therapy Alters the Course of Recurrent Multidrug-Resistant Urinary Tract Infections

**DOI:** 10.3390/life15010050

**Published:** 2025-01-02

**Authors:** Alexandru Ciudin, Bernat Padulles, Pasqualino Manasia, Josep Alcoberro, Cristian Toma, Răzvan Popescu, Cristian Persu, Antonio Aguilar

**Affiliations:** 1 Urology Department, Hospital Universitari de Mollet, 08100 Barcelona, Spain; b.padulles@fsm.cat (B.P.); p.manasia@fsm.cat (P.M.); j.alcoberro@fsm.cat (J.A.); an.aguilar@fsm.cat (A.A.); 2 Urology Department, Spitalul Clinic “Prof. Dr. Th. Burghele”, 061344 Bucuresti, Romania; razvan-ionut.popescu@drd.umfcd.ro (R.P.); cristian.persu@umfcd.ro (C.P.)

**Keywords:** autovaccine therapy, multidrug-resistant bacteria, urinary tract infections (UTIs), antibiotic resistance, personalized medicine, bacterial immunotherapy, infection recurrence, healthcare resource utilization, antibiotic stewardship, sublingual vaccination

## Abstract

Background/Objectives: Urinary tract infections (UTIs) caused by multidrug-resistant (MDR) bacteria pose a considerable challenge due to high treatment failure rates and associated healthcare costs. This pioneering study evaluates the effectiveness of personalized autovaccine therapy in managing recurrent UTIs in patients with MDR bacteria, aiming to offer an innovative treatment that reduces antibiotic resistance and hospitalizations. Methods: In this prospective, single-center study, 40 patients with recurrent MDR UTIs received personalized sublingual autovaccines derived from their own bacterial isolates. The study assessed UTI recurrence rates, changes in antibiotic use, and hospitalization days over 12 months. Results: The autovaccine therapy significantly reduced UTI recurrence, with 67.5% of patients experiencing fewer infections. Antibiotic usage decreased by 74.4%, and total hospitalization days annually reduced from 400 to 216. A significant shift was observed from MDR to multi-susceptible bacterial profiles among participants. Conclusions: This study is the first to demonstrate that autovaccine therapy is a safe and effective approach for managing recurrent UTIs caused by MDR bacteria, significantly lowering infection frequency, antibiotic needs, and hospitalization. These findings support integrating autovaccine therapy into standard UTI management to combat antibiotic resistance and lessen healthcare burdens.

## 1. Introduction

Recurrent urinary tract infections (UTIs) caused by multidrug-resistant (MDR) bacteria are a pressing global health concern, associated with increased morbidity, prolonged hospital stays, and significant economic burdens due to escalating healthcare costs [1,2,3,4]. These infections often result from the overuse and misuse of antibiotics, leading to the emergence of resistant bacterial strains, which render traditional therapies less effective [5,6]. Consequently, alternative therapeutic strategies that bypass the limitations of conventional antibiotics are urgently needed [4,7].

Autovaccine therapy has emerged as a promising solution, offering a personalized approach to managing recurrent UTIs. These vaccines are derived from the patient’s own bacterial isolates and are designed to stimulate both mucosal and systemic immune responses, enhancing the body’s ability to recognize and combat specific pathogens [8,9]. Unlike broad-spectrum antibiotics, which disrupt the natural microbiota and contribute to resistance, autovaccines provide targeted immunity while preserving the host’s microbial balance [8,10,11].

Vaccines have long been employed to prevent recurrent UTIs. Initially, they consisted of mixtures of bacterial strains, beginning exclusively with *E. coli* strains (OM-89) [12]. Over time, these vaccines advanced to combined formulations that included fixed proportions of various bacterial strains or even allowed for flexible combinations tailored to the patient’s prior urine culture results. The development of autovaccines marked the culmination of this evolution, utilizing the patient’s own bacterial strains rather than standardized collections, thus enabling a highly personalized approach to managing recurrent UTIs [4]. Studies have demonstrated the efficacy of autovaccines in reducing UTI recurrence rates and improving quality of life in patients with recurrent infections [3,8,9,13,14,15,16].

Autovaccines for recurrent UTIs are not designed as a treatment for active infections but rather as a prophylactic measure. By proactively stimulating the immune system, these vaccines aim to reduce the frequency of infections, aligning with the principle of prevention over cure. Administered over a typical period of three months, they have been shown to significantly decrease the number of infectious episodes, thereby improving patients’ quality of life by reducing the physical and emotional burden of recurrent infections. Additionally, vaccines provide substantial economic benefits by lowering healthcare costs [14,17,18]. Studies comparing their use with prophylactic antibiotics demonstrated reduced antibiotic consumption and fewer medical consultations, emergency visits, and diagnostic tests. This dual impact—enhancing patient well-being and decreasing healthcare expenditure—highlights the value of vaccines as a sustainable approach to managing recurrent UTIs, with average annual costs per patient dropping more than 500 EUR [19].

Patients with recurrent UTIs caused by MDR bacteria represent a population with high healthcare resource utilization. These patients often require prolonged intravenous antibiotic therapy, administered either through conventional hospitalization or home-based care. While this approach is essential for managing severe infections, it imposes a substantial economic burden on healthcare systems and severely impacts patients’ quality of life. The need for devices such as intravenous catheters, frequent visits from healthcare personnel, and the physical and social limitations associated with extended treatment regimens contribute to a debilitating experience. This highlights the critical need for effective alternatives that not only reduce infection recurrence but also minimize reliance on intensive healthcare resources. The extra healthcare costs and productivity losses due to multidrug-resistant bacteria in the EU has been evaluated to a staggering EUR 1.5 billion each year [20,21]. There are currently no studies in the literature that specifically evaluate the role of immunotherapy through autovaccines in reducing infection rates in patients with MDR infections.

This is the first study to evaluate the effectiveness of personalized autovaccine therapy in patients with recurrent MDR UTIs. It focuses on recurrence rates, changes in bacterial resistance profiles, and the reduction in healthcare resource utilization. By addressing these gaps, the research aims to provide valuable insights into the role of autovaccines as a sustainable and innovative solution in the management of MDR UTIs.

## 2. Materials and Methods

### 2.1. Study Design

This research was designed as a prospective, single-center observational study. Patients were closely analyzed between August 2018 and August 2023 to evaluate the efficacy and safety of autovaccine therapy in patients with recurrent urinary tract infections (UTIs) caused by multidrug-resistant (MDR) bacteria. The study followed a 12-month follow-up design to assess the impact of personalized autovaccine therapy on UTI recurrence rates, bacterial resistance profiles, antibiotic use, and healthcare resource utilization.

Urine collection was performed in compliance with International Safety Standards [22], using sterile receptacles for culturing on CHROMID^®^ CPS^®^ Elite Agar (CPSE) (bioMérieux, Madrid, Spain) [23]. The incubation of these cultures lasted between 24 and 48 h at 37 °C, focusing solely on pure cultures, with a threshold of 105 CFU/mL considered positive for gram-negative bacilli. Microorganism identification and antibiotic susceptibility testing were performed by the automated MicroScan Walkway 96 (Beckman-Coulter, Madrid, Spain); additionally, if necessary, the Kirby-Bauer diffusion disk method was used. Minimum inhibitory concentrations (MICs) and zone diameter breakpoints were interpreted according to criteria established by EUCAST guidelines [24].

Inclusion Criteria:

- Adult patients aged >18 years.
- History of recurrent UTIs, defined as:
  ○ Three or more symptomatic episodes in the previous 12 months, or
  ○ Two or more symptomatic episodes in the last six months [4].
- Positive urine cultures identifying MDR uropathogens, defined as >10^5^ UFC bacteria resistant to oral antibiotics, including extended-spectrum beta-lactamase (ESBL) producing organisms.

Exclusion Criteria:

- Less than 18 years of age.
- Pregnancy or breastfeeding.
- Significant urological abnormalities requiring surgical intervention (e.g., bladder stones, severe pelvic organ prolapse).
- Immunocompromised conditions, such as HIV/AIDS or ongoing immunosuppressive therapy.
- History of severe allergic reactions to vaccines or vaccine components.
- Participation in another clinical trial within the last 30 days.
- Patients unable or unwilling to adhere to the study protocol.

### 2.2. Intervention

Patients received a personalized sublingual autovaccine prepared from their own isolated uropathogens. The process involved the following steps:

1. Sample Collection:
  Midstream urine samples were collected during an active symptomatic UTI episode. Samples were transported to a certified microbiological laboratory under sterile conditions for analysis.
2. Pathogen Identification:
  Bacteria were cultured, isolated, and tested for antimicrobial resistance to confirm MDR status. Pathogens were further identified to the species level using standard microbiological techniques.
  The identification of gram-positive and gram-negative bacteria was achieved using both phenotypic and molecular methods to ensure comprehensive and accurate results. Initially, standard phenotypic methods were employed, which included:
    1. Cultural Characteristics: Bacteria were cultured on selective and differential media to observe colony morphology, color, and hemolytic properties.
    2. Biochemical Testing: Standard tests such as catalase, coagulase (for gram-positive cocci), oxidase, and API systems (for gram-negatives) were utilized to determine biochemical properties that distinguish different bacterial genera and species.
    3. Antimicrobial Susceptibility Patterns: The Kirby-Bauer disk diffusion method provided additional phenotypic data relevant to bacterial identification.
  Antimicrobial susceptibility testing was conducted to evaluate the efficacy of various antimicrobials against isolated pathogens, essential for managing MDR urinary tract infections. The updated antimicrobial panel included:
    1. Beta-lactams: Ampicillin, Amoxicillin–Clavulanic Acid (Amoxi–Clav), Piperacillin–Tazobactam (Piper–Tazo), Ertapenem, Meropenem
    2. Cephalosporins: Representative agents from second, third, and fourth generations
    3. Fluoroquinolones: Ciprofloxacin
    4. Aminoglycosides: Gentamicin, Amikacin
    5. Others: Trimethoprim/Sulfamethoxazole, Nitrofurantoin, Fosfomycin, Colistin
  These choices cover a broad spectrum of mechanisms of action and are critical for a comprehensive assessment of bacterial susceptibility and resistance patterns. Minimum inhibitory concentrations (MICs) and zone diameter breakpoints were interpreted according to criteria established by EUCAST guidelines.
  This detailed inclusion ensures that the study methods are thoroughly described, aligning with rigorous scientific standards and enhancing the manuscript’s credibility. The inclusion of a wide range of antimicrobials allows for an extensive analysis of resistance trends, crucial for advancing treatment strategies against MDR infections.
3. Vaccine Preparation:
  The isolated bacteria were inactivated, lysed, and processed into a sublingual solution containing 2000 million germ equivalents per milliliter. The preparation was performed by a certified laboratory (Probelte Pharma, Murcia, Spain) in compliance with Good Manufacturing Practices (GMP). While the general process of autovaccine production is summarized here, the specific steps involved are proprietary and cannot be disclosed in detail due to patent protections. However, it is important to note that the autovaccine is approved by Spanish health authorities and meets all the GMP standards for autovaccine production.
4. Administration Protocol:
  Patients initiated treatment with one drop of autovaccine per day, progressively increasing to five drops daily over the first week. The regimen was maintained for three months. Repeat courses were allowed if clinically indicated based on follow-up results.

Follow-Up and Data Collection
  - Participants were evaluated at baseline and at 3, 6, and 12 months post-treatment initiation. The following data were collected at each visit:
  - Clinical Assessments: Symptoms of UTIs, including dysuria, frequency, urgency, and hematuria, were recorded.
  - Urine Cultures: Samples were analyzed to monitor bacterial profiles, including the persistence or eradication of MDR bacteria and any transition to multi-susceptible strains.
  - Antibiotic Use: Data on all antibiotic treatments administered during the study period were recorded.
  - Adverse Events: Patients were asked to report any adverse effects, which were categorized as mild, moderate, or severe.
  - Patients maintained diaries to record symptoms, medication usage, and any side effects during the study period.
Outcome Measures
  1. The primary outcome was the reduction in UTI recurrence rates over 12 months compared to baseline.
  2. The secondary outcomes were:
    a. Time to First Recurrence: Time elapsed between the start of therapy and the first symptomatic UTI.
    b. Negativization of Cultures: Proportion of patients achieving negative urine cultures at 3, 6, and 12 months.
    c. Bacterial Resistance Profiles: Proportion of patients transitioning from MDR to multi-susceptible (MULTI-S) bacterial strains.
    d. Antibiotic usage: Reduction in the total number of antibiotic courses required for UTI management.
    e. Safety and Tolerability: Incidence and severity of adverse events related to autovaccine therapy.
    f. Hospitalization: Reduction in the number of inpatient or home-based antibiotic therapy days.

### 2.3. Statistical Analysis

Data were analyzed using descriptive and inferential statistics. Continuous variables (e.g., number of UTI episodes, time to first recurrence) were compared using paired *t*-tests or Wilcoxon signed-rank tests. Categorical variables (e.g., bacterial resistance profiles, culture negativization rates) were analyzed using chi-square or Fisher’s exact tests. Cox proportional hazards models were employed to estimate the hazard ratios for UTI recurrence. Statistical significance was defined as *p* < 0.05. Statistical analyses in this study were conducted using SPSS 16.0.

### 2.4. Sample Size Calculation

The study was powered to detect a 40% reduction in UTI recurrence rates with a significance level (α) of 0.05 and a power (1 − β) of 80%. Allowing for a 10% dropout rate, 40 patients were enrolled to ensure adequate statistical power.

## 3. Results

### 3.1. Study Population

A total of 40 patients (82.5% women) aged between 32 and 75 years were included in the study. All participants had a history of recurrent urinary tract infections (UTIs) caused by multidrug-resistant (MDR) bacteria. Before autovaccine therapy, patients experienced an average of 4.2 UTI episodes per year, each requiring intravenous antibiotic treatment. The average time to recurrence prior to the intervention was 3.0 months.

### 3.2. Reduction in UTI Recurrence Rates

Autovaccine therapy led to a significant reduction in UTI recurrence rates over the 12-month follow-up period. At the baseline, all patients had had at least three UTI episodes in the preceding year, with a cumulative total of 168 episodes among the cohort. At the 12-month mark, the total number of UTI episodes had decreased to 61, reflecting a 63.7% reduction. Among the 40 patients, 27 (67.5%) achieved a clinically significant reduction in UTI recurrence, with 22 of them remaining recurrence-free. Importantly, the mean number of UTIs per patient decreased significantly from 4.2 at baseline to 1.525 after treatment (*p* < 0.001) (Table 1).

Patients who recurred experienced fewer episodes, with a shift towards less severe infections. Of the symptomatic UTIs recorded at 12 months, seven episodes were caused by MDR bacteria, while six were associated with multi-susceptible strains. Notably, no patient transitioned from multi-susceptible strains back to MDR profiles during the study.

### 3.3. Bacterial Resistance and Profile Evolution

A key finding of this study was the significant shift in bacterial resistance profiles. At baseline, all 40 patients presented with MDR pathogens. The bacterial isolates identified in our study exhibited resistance to a broad spectrum of antibiotics commonly employed in the management of urinary tract infections. Resistance was noted across several major classes of antibiotics, including beta-lactams such as penicillins (ampicillin), cephalosporins (e.g., cefuroxime, ceftriaxone, cefepime). Additionally, resistance extended to fluoroquinolones like ciprofloxacin and levofloxacin; aminoglycosides, including gentamicin and amikacin; and sulfonamides such as trimethoprim/sulfamethoxazole. Other classes showing resistance were tetracyclines (e.g., doxycycline), glycopeptides (e.g., vancomycin, primarily against gram-positive bacteria), and macrolides (e.g., erythromycin). Isolates also showed resistance to fosfomycin, nitrofurantoin, and colistin. These findings were determined through standardized antimicrobial susceptibility testing using microdilution (MicroScan Walkaway96) [23] and the Kirby-Bauer disk diffusion method, interpreted according to EUCAST guidelines [24]. The diverse resistance profiles highlight the need for precise antimicrobial stewardship in treating infections caused by these resistant pathogens.

By 12 months, 26 patients (65%) had transitioned to MULTI-S bacterial strains, and 22 achieved culture-negative status. This gradual improvement was evident across all follow-up time points, with culture-negative patients increasing from 0 at baseline to 24 at 3 months, 28 at 6 months, and 22 at 12 months.

Changes in bacterial species were also observed. At baseline, *E. coli* and *K. pneumoniae* were the predominant pathogens, accounting for 90% of MDR cases. By 12 months, among the 26 patients who transitioned to multi-susceptible profiles, most retained the same species with altered resistance. Specifically, 65% of *E. coli* strains and 50% of *K. pneumoniae* strains transitioned from MDR to multi-susceptible. For the remaining patients, bacterial shifts occurred, including transitions to *Enterococcus* spp., a less virulent species frequently associated with asymptomatic bacteriuria. No *Proteus mirabilis* or *Pseudomonas aeruginosa* strains were detected in follow-up cultures, reflecting a potential eradication of these pathogens post-treatment (Table 2 and Table 3).

### 3.4. Time to First Recurrence

The mean time to first recurrence significantly increased from 3.0 months before treatment to 3.9 months after treatment (*p* < 0.001). Patients who remained recurrence-free at 12 months demonstrated a robust immune response attributed to the autovaccine. Those who recurred experienced a longer interval between episodes compared to their pre-treatment history.

### 3.5. Antibiotic Use

A substantial reduction in antibiotic consumption was observed following autovaccine therapy. At baseline, all 40 patients required antibiotics for each UTI episode. After treatment, only 23 patients required antibiotics, representing a 42.5% decrease in antibiotic dependence. Among these, 16 patients required intravenous antibiotics to manage MDR infections, whereas the remaining patients were treated with oral antibiotics for less resistant strains (Table 4). This change reflects not only the reduced number of UTIs but also a shift towards treating less severe infections.

### 3.6. Hospitalization and Healthcare Resource Utilization

The total number of hospitalization days decreased significantly following autovaccine therapy. Without the intervention, the estimated hospital days for managing recurrent UTIs would have been 400. Post-treatment, the actual number of hospitalization days was reduced to 216, reflecting a 184-day reduction. Notably, among patients who recurred with MDR bacteria, 40% required inpatient hospitalization, while the remaining 60% were managed with home-based intravenous therapy. These findings highlight the potential of autovaccine therapy to reduce the burden on healthcare systems.

### 3.7. Safety and Tolerability

No severe adverse events were reported during the study. Mild side effects, such as an unpleasant taste of the sublingual solution, were noted but did not lead to treatment discontinuation. Overall, the therapy was well-tolerated by the majority of participants.

### 3.8. Summary of Bacterial Changes

The study demonstrated not only a reduction in MDR pathogens but also a shift in bacterial species over the course of 12 months. At baseline, *E. coli* accounted for 45% of isolates, followed by *K. pneumoniae* (40%), *Proteus mirabilis* (5%), *Pseudomonas aeruginosa* (5%), and *Enterococcus* spp. (5%). By 12 months, *E. coli* and *K. pneumoniae* remained dominant among multi-susceptible strains, but with reduced virulence. *Enterococcus* spp. increased to 20% of isolates, while *Proteus mirabilis* and *Pseudomonas aeruginosa* were eradicated from the cohort. This shift in bacterial profiles likely reflects both the targeted effect of the autovaccine and improved host immune defence abilities.

## 4. Discussion

Recurrent urinary tract infections (UTIs) caused by MDR bacteria remain a significant challenge in clinical practice. These infections are associated with limited treatment options, increased morbidity, and significant economic burdens due to prolonged hospital stays and higher healthcare costs. The rise in antimicrobial resistance further complicates the management of these infections, as many conventional antibiotics lose their efficacy [4,11,20,25,26]. Consequently, innovative therapeutic strategies are urgently needed to address this pressing issue.

This study is the first to evaluate the effectiveness of personalized autovaccine therapy in managing recurrent urinary tract infections (UTIs) caused by MDR bacteria. Unlike previous research focusing on standard antibiotic therapies or broader immunotherapeutic approaches, this investigation specifically addresses a critical gap: the impact of tailored immunotherapy on patients with infections resistant to multiple antibiotic classes. Personalized autovaccines, derived from the patient’s own bacterial isolates, represent a novel therapeutic strategy that harnesses the immune system’s ability to recognize and combat specific pathogens.

By targeting MDR bacteria, this study explores a promising alternative to traditional treatments, which are often limited in efficacy and associated with significant side effects, including the development of further resistance. The unique approach of this study lies in its focus on a highly challenging patient population, often requiring prolonged intravenous antibiotic therapy and hospitalization, which greatly impacts their quality of life and healthcare resources.

Our findings demonstrate that autovaccine therapy significantly reduces UTI recurrence rates, with a 67.5% decrease in recurrent episodes at 12 months. These results align with previous studies showing reductions in recurrence rates ranging from 60% to 75% among patients treated with autovaccines [3,4,8,9,13,14]. Additionally, the time to first recurrence increased significantly from an average of 3.0 months to 3.9 months post-treatment, indicating sustained protection. This extended recurrence-free interval is particularly relevant for patients with MDR infections, as it reduces their exposure to antibiotics and lowers the likelihood of developing resistance to additional drug classes [11,26].

The personalized nature of autovaccine therapy may explain its efficacy. Unlike broad-spectrum antibiotics, autovaccines are tailored to target the specific pathogens responsible for each patient’s infections, leveraging the immune system to achieve a precise and robust response. By introducing inactivated bacterial components, autovaccines stimulate both mucosal and systemic immunity, which may explain the observed long-term reduction in recurrence rates [27,28].

The reduction in recurrence rates also translated into significant reductions in antibiotic use. At baseline, all 40 patients required antibiotics for UTI episodes. Post-intervention, only 23 patients required antibiotics, representing a 42.5% decrease in usage. Moreover, the nature of the infections shifted, with fewer MDR-related episodes, allowing the use of narrower-spectrum antibiotics in some cases. These findings align with reports that show that autovaccine therapy not only reduces infection rates but also minimizes reliance on antibiotics [4,8,9,13,15,20,25,27,29].

This reduction in antibiotic use is crucial for combating the global crisis of antimicrobial resistance. Excessive antibiotic use is a primary driver of resistance, and interventions that limit their usage, like autovaccine therapy, are urgently needed to preserve antibiotic efficacy [20,21]. Furthermore, the reduction in hospitalization days from an estimated 400 days to 216 days reflects the broader impact of autovaccine therapy on healthcare resource utilization. By reducing the frequency and severity of infections, this approach alleviates the burden on healthcare systems, lowers costs, and improves patient quality of life.

Among patients with recurrent infections, 40% required traditional inpatient care, while 60% were managed successfully with home-based intravenous therapy. This shift highlights the reduced severity and frequency of infections, enabling less resource-intensive management approaches.

Hospital stays, often driven by the need for prolonged intravenous antibiotic administration, are among the most costly components of managing recurrent MDR UTIs. Reducing hospitalizations not only minimizes direct costs but also alleviates pressure on healthcare systems, freeing up resources for other critical cases. These findings underline the economic and clinical advantages of autovaccine therapy in optimizing healthcare resource allocation [30,31].

MDR infections significantly increase healthcare costs and are associated with extended hospital stays and high mortality rates. Studies indicate that nosocomial infections caused by MDR bacteria contribute to delayed therapies and higher treatment failures. In the European Union alone, MDR infections result in 25,000 annual deaths and €1.5 billion in additional healthcare costs [20,21,31]. By reducing infection recurrence and shifting bacterial resistance profiles, autovaccine therapy addresses these challenges effectively.

One of the most notable findings of this study was the transition of bacterial profiles in patients with MDR infections. At baseline, all patients harbored MDR pathogens, primarily *E. coli* and *K. pneumoniae*. By the 12-month follow-up, 65% of these patients had transitioned to MULTI-S bacterial profiles, and 55% achieved culture-negative status. These results suggest that autovaccine therapy not only reduces recurrence, but may also influence bacterial resistance patterns [20,25,29].

The observed shift from MDR bacteria to MULTI-S strains in this study can be explained by several mechanisms related to the action of autovaccine immunotherapy and the host-microorganism dynamics. The first one would be immune system stimulation. Autovaccines enhance both systemic and mucosal immunity, boosting the host’s ability to identify and eliminate specific pathogenic bacteria. This improved immune response may reduce the initial MDR bacterial load [9,13,25], allowing less resistant and less adapted strains to dominate.

Another mechanism could be the reduction of selective pressure. By reducing the use of broad-spectrum antibiotics, the selective pressure that favors the survival and proliferation of MDR strains is diminished. This allows more susceptible bacteria to replace resistant strains [32]. Finally, another hypothesis is the decreased virulence of MDR bacteria. MDR bacteria often incur fitness costs due to the metabolic burden of maintaining complex resistance mechanisms. When the selective pressure from antibiotics is removed, less resistant but more adaptive strains can outcompete MDR bacteria [33,34,35].

The primary goal of evaluating changes in resistance patterns was to determine whether autovaccine therapy could restore susceptibility to oral antibiotics, thereby providing clinicians with more treatment options. Shifts from MDR to MULTI-S profiles were particularly relevant when they indicated a return of sensitivity to oral antibiotics such as fluoroquinolones or cephalosporins. These changes directly impacted the feasibility of transitioning patients to oral regimens and reducing reliance on intravenous antibiotics.

In cases where MDR status changed due to altered sensitivity to intravenous antibiotics, such as amikacin, these changes were not considered clinically significant within the scope of our study. From a practical standpoint, resistance to intravenous antibiotics does not alter the patient’s immediate treatment options, as they would still require IV therapy regardless of susceptibility changes in this category. Thus, our study emphasized the relevance of changes in oral antibiotic resistance, reflecting our focus on improving outpatient treatment strategies and reducing the burden of intravenous therapies.

This shift is particularly significant because it demonstrates that autovaccine therapy not only reduces infection recurrence but also modulates bacterial ecology toward more manageable and less resistant profiles. These findings underscore the comprehensive impact of autovaccines on bacterial resistance dynamics and patient health.

Additionally, the changes in bacterial species observed during the study highlight another potential benefit of autovaccine therapy. While *E. coli* and *K. pneumoniae* remained dominant among multi-susceptible strains, Proteus mirabilis and Pseudomonas aeruginosa were no longer detected in follow-up cultures. *Pseudomonas aeruginosa* and *Proteus mirabilis* are highly virulent pathogens but often incur significant metabolic costs to maintain their resistance and pathogenicity mechanisms. These fitness costs may make them less competitive in the absence of antibiotic pressure and in an environment where the immune response has been enhanced [36,37,38]. The immune modulation induced by autovaccines may alter the urinary microbiota, creating conditions unfavorable for *Proteus mirabilis* and *Pseudomonas aeruginosa*. These species often thrive in specific niches, such as environments with higher urea concentrations or disrupted microbiota [39,40]. Restoring balance in the microbiota could indirectly suppress their growth.

Autovaccine therapy was well-tolerated by all patients, with no severe adverse events reported. Mild side effects, such as the unpleasant taste of the sublingual solution, were noted, but did not lead to treatment discontinuation. This excellent safety profile is consistent with prior studies evaluating autovaccine use in recurrent UTIs, reinforcing its suitability for long-term management of this patient population [3,8,9,13,14,15].

The absence of serious adverse events is particularly noteworthy, as it underscores the potential of autovaccine therapy as a safe alternative to traditional antibiotic regimens, which often carry a higher risk of adverse effects [26,41,42,43,44,45].

Despite the promising results, this study has several limitations that must be acknowledged. The sample size of 40 patients is relatively small, which may limit the possibility of extrapolating the findings. Additionally, the absence of a control group prevents direct comparisons with other treatment modalities, such as continuous antibiotic prophylaxis or other forms of immunotherapy. Further research, including randomized controlled trials with larger cohorts, is necessary to confirm these findings and evaluate the long-term impact of autovaccine therapy on antimicrobial resistance patterns.

Future studies should focus on optimizing autovaccine production and administration protocols to enhance efficacy and scalability. Investigations into the mechanisms underlying bacterial profile transitions could provide valuable insights into how autovaccines influence resistance dynamics. Moreover, exploring the integration of autovaccines with other novel therapies, such as bacteriophage therapy, could yield synergistic effects and improve outcomes for patients with complex infections [46].

## 5. Conclusions

Autovaccine therapy represents a safe and effective strategy for managing recurrent MDR UTIs. By significantly reducing recurrence rates, antibiotic use, and hospitalizations, while promoting favorable shifts in bacterial resistance profiles, autovaccine therapy addresses both patient-level and systemic challenges associated with MDR infections. These findings support the broader adoption of autovaccine therapy in clinical practice and highlight its potential to mitigate the growing threat of antimicrobial resistance.

## Figures and Tables

**Table 1:** Changes in UTIs and bacterial profiles over time.

Timepoint	Total UTIs (Episodes)	Culture-Negative (%)	MDR (%)	Multi-Susceptible (%)
Baseline	168	0	100.0	0.0
3 Months	72	55	37.5	7.5
6 Months	48	70	30.0	12.5
12 Months	61	55	17.5	35.0

**Table 2:** Bacterial species evolution over 12 months.

Bacterial Species	Baseline MDR (n)	12 Months MDR (n)	12 Months Multi-S (n)	12 Months Culture-Negative (n)
*E. coli*	18	8	8	2
*K. pneumoniae*	16	4	4	8
*Proteus mirabilis*	2	0	0	2
*Pseudomonas aeruginosa*	2	0	0	2
*Enterococcus* spp.	2	1	1	1

**Table 3:** Evolution of patients and bacterial profiles.

Patient ID	Baseline UTIs	Baseline Bacterial Profile	12 Month UTIs	12-Month Bacterial Profile
1	3	*K. pneumoniae MDR*	0	*K. pneumoniae MULTI-S*
2	5	*K. pneumoniae MDR*	1	*K. pneumoniae MULTI-S*
3	5	*E. coli MDR*	3	*E. coli MULTI-S*
4	5	*K. pneumoniae MDR*	1	*K. pneumoniae MULTI-S*
5	5	*E. coli MDR*	2	*E. coli MULTI-S*
6	4	*K. pneumoniae MDR*	3	*K. pneumoniae MULTI-S*
7	5	*E. coli MDR*	1	*E. coli MULTI-S*
8	5	*E. coli MDR*	0	*K. pneumoniae MULTI-S*
9	5	*E. coli MDR*	1	*E. coli MULTI-S*
10	5	*K. pneumoniae MDR*	4	*E. coli MULTI-S*
11	4	*K. pneumoniae MDR*	2	*Enterococcus* spp. *MULTI-S*
12	5	*K. pneumoniae MDR*	1	*E. coli MULTI-S*
13	4	*K. pneumoniae MDR*	4	*E. coli MULTI-S*
14	3	*E. coli MDR*	0	*E. coli MULTI-S*
15	5	*K. pneumoniae MDR*	1	*E. coli MULTI-S*
16	4	*E. coli MDR*	2	*Enterococcus* spp. *MULTI-S*
17	4	*E. coli MDR*	1	*K. pneumoniae MULTI-S*
18	5	*K. pneumoniae MDR*	3	*Enterococcus* spp. *MULTI-S*
19	4	*E. coli MDR*	0	*K. pneumoniae MULTI-S*
20	3	*K. pneumoniae MDR*	2	*K. pneumoniae MDR*
21	5	*E. coli MDR*	1	*E. coli MDR*
22	5	*K. pneumoniae MDR*	1	*K. pneumoniae MDR*
23	4	*K. pneumoniae MDR*	2	*K. pneumoniae MDR*
24	3	*K. pneumoniae MDR*	1	*K. pneumoniae MDR*
25	5	*E. coli MDR*	3	*E. coli MDR*
26	5	*E. coli MDR*	1	*E. coli MDR*
27	3	*K. pneumoniae MDR*	2	*K. pneumoniae MDR*
28	3	*K. pneumoniae MDR*	0	*K. pneumoniae MDR*
29	3	*K. pneumoniae MDR*	1	*K. pneumoniae MDR*
30	3	*E. coli MDR*	3	*E. coli MDR*
31	5	*E. coli MDR*	2	*E. coli MDR*
32	3	*E. coli MDR*	1	*E. coli MDR*
33	4	*K. pneumoniae MDR*	3	*K. pneumoniae MDR*
34	4	*K. pneumoniae MDR*	1	*K. pneumoniae MDR*
35	5	*K. pneumoniae MDR*	0	*K. pneumoniae MDR*
36	4	*E. coli MDR*	2	*E. coli MDR*
37	4	*E. coli MDR*	1	*E. coli MDR*
38	3	*E. coli MDR*	3	*E. coli MDR*
39	5	*E. coli MDR*	1	*E. coli MDR*
40	4	*E. coli MDR*	0	*E. coli MDR*

MDR—multidrug-resistant. MULTI-S—multi-sensible.

**Table 4:** Antibiotic use and hospitalization metrics.

Metric	Baseline	Post-Treatment	Reduction (%)
Total Antibiotic Courses (patients)	40	23	42.5
Intravenous Antibiotic Courses (patients)	40	16	60.0
Total Hospitalization Days	400	216	54.0

## Data Availability

The data are not available due to the Spanish Organic Lawon Data Protection.
